# Common Carotid Artery Extra-Media Thickness as a Marker of Perivascular Obesity and Cardiometabolic Risk in Individuals Without Cardiovascular Disease

**DOI:** 10.14740/jocmr6657

**Published:** 2026-09-26

**Authors:** Anna Bragina, Natalia Druzhinina, Yulia Rodionova, Daria Akvitskaya, Kirill Novikov, Galina Nikiforova, Oksana Avdeenko, Natalia Vlasova, Alina Arzukanyan, Alina Kudravceva, Valery Podzolkov

**Affiliations:** a Department of Internal Medicine No. 2, Institute of Clinical Medicine, Sechenov First Moscow State Medical University, 19991 Moscow, Russia; b Department for Ear, Nose and Throat Diseases, Institute of Clinical Medicine, Sechenov First Moscow State Medical University, 19991 Moscow, Russia; c Department of Therapeutic Dentistry, Institute of Dentistry Named After E.V. Borovsky, Sechenov First Moscow State Medical University, 19991 Moscow, Russia

**Keywords:** Adipose tissue, Extra-media thickness, Perivascular obesity, Carotid arteries, Obesity, Risk factors

## Abstract

**Background:**

Perivascular adipose tissue (PVAT) is increasingly recognized as a pathogenetic contributor to vascular dysfunction. Carotid extra-media thickness (EMT) measured by ultrasound is the simplest, noninvasive and clinically accessible marker of PVAT deposition. However, reference values in young individuals without cardiovascular disease (CVD) are lacking. The aim of this study was to analyze the relationship between carotid EMT and cardiovascular risk factors and to determine a tentative reference value of carotid EMT in individuals without CVD.

**Methods:**

This cross-sectional study included 373 patients with a median age of 23 (21; 32) years and without CVD. All patients underwent anthropometric examination, lipid-profile assessment using the CardioChek PA (USA, 2020), and duplex ultrasound of the brachiocephalic arteries with measurement of common carotid artery EMT and intima-media thickness (IMT). A subgroup of apparently healthy individuals without cardiovascular risk factors was selected to define a threshold. EMT above the 90th percentile of this subgroup was regarded as perivascular obesity (PVO).

**Results:**

Carotid EMT was significantly associated with age, blood pressure levels, anthropometric and laboratory metabolic markers, glycemia and smoking. On multivariable linear regression, age, obesity and smoking were independently associated with EMT. The 90th percentile threshold in the apparently healthy subgroup (n = 185) was 0.54 mm; EMT ≥ 0.54 mm (PVO) was identified in 24.2% of the sample and was most frequent in individuals aged 41–55 years. On multivariable logistic regression, age and waist circumference were independently associated with the presence of PVO.

**Conclusions:**

Carotid EMT, reflecting the severity of PVAT deposition, may be associated with a wide range of cardiometabolic risk factors in individuals without CVD, with a tentative threshold value of 0.54 mm.

## Introduction

Obesity represents a significant medical and social problem worldwide [1], with a high prevalence not only in older adults [2] but also in younger age groups [3, 4]. Although the rate of increase in obesity prevalence has slowed in several high-income countries [5], the global burden of obesity and associated cardiovascular complications continues to rise [2].

Obesity is characterized by a variety of phenotypes [6]. Some of these phenotypes may evolve over time into a metabolically unhealthy phenotype with increased cardiovascular risk [7]. The variability of adipose tissue distribution is well recognized and is partly related to the formation of ectopic fat depots [8–10]. Various visceral fat depots, including pararenal adipose tissue (PRAT) [11], pericardial adipose tissue (PAT) [12], and perivascular adipose tissue (PVAT) [13], are considered potential contributors to dysfunction of the organs they surround [14]. With excessive accumulation, PVAT may develop a dysfunctional phenotype and synthesize a broad spectrum of adipokines that contribute to meta-inflammation, vascular remodeling, increased vascular stiffness, atherosclerosis, and thrombosis [13–15].

For PVAT imaging, several consensus documents recommend ultrasound, magnetic resonance imaging, multislice computed tomography (MSCT), and positron emission tomography-computed tomography [16]. We have previously proposed tentative reference values for periaortic adipose tissue based on chest MSCT data [17]. However, the use of radiological methods may be limited by radiation exposure, cost, and accessibility. In this regard, ultrasound of the brachiocephalic arteries may be considered a clinically accessible approach for evaluating PVAT. Ultrasound measurement of common carotid artery extra-media thickness (EMT) has been proposed as a simple, noninvasive, and clinically available marker of PVAT [17]. The extra-media complex comprises the arterial adventitia and the PVAT surrounding the vessel [18].

An association of EMT with obesity and metabolic syndrome (MS) has been demonstrated in elderly patients [19]. It has been suggested that EMT, unlike intima-media thickness (IMT), may reflect not only vascular wall stiffness but also PVAT accumulation as a potential pathogenetic contributor to vascular dysfunction [19]. A meta-analysis demonstrated a significant relationship between PVAT, assessed by ultrasound and radiological methods, and hypertension, dyslipidemia, and obesity markers, including body mass index (BMI), waist circumference (WC) [20, 21]. Nevertheless, data on reference values of carotid EMT as an accessible marker of PVAT in young individuals without cardiovascular disease (CVD) remain limited.

Accordingly, the aim of our study was to analyze the relationship between carotid EMT and cardiovascular risk factors and to determine a tentative reference value of carotid EMT in individuals without CVD.

## Materials and Methods

This cross-sectional study was conducted at University Clinical Hospital No. 4 of Sechenov University in accordance with the ethical principles of the Declaration of Helsinki. The study was approved by the local ethics committee on December 8, 2022 (protocol No. 25–22). All participants provided written informed consent. The inclusion criteria were an age between 18 and 55 years and consent to participate in the study.

The exclusion criteria were as follows: presence of symptomatic hypertension; clinical manifestations of CVD, including coronary heart disease and cerebrovascular disease; presence of atherosclerotic plaques; clinical and laboratory manifestations of chronic liver disease; decreased glomerular filtration rate < 60 mL/min/1.73 m^2^; proteinuria ≥ 300 mg/day; type 1 or type 2 diabetes mellitus; inflammatory or oncological diseases of any localization; pregnancy at the time of inclusion; and use of medications for the treatment of obesity or dyslipidemia.

The study included 373 patients with a median age of 23 (21; 32) years (Table 1). History data on the presence and duration of hypertension and smoking status were collected. All patients underwent anthropometric examination with measurement of WC, hip circumference (HC), and neck circumference. BMI was calculated as weight divided by height squared (kg/m^2^). Biochemical lipid-profile parameters, including total cholesterol (TC), low-density lipoprotein cholesterol (LDL-C), high-density lipoprotein cholesterol (HDL-C), and triglycerides (TG), were assessed using a CardioChek PA express analyzer (USA, 2020). Overweight and obesity grade were assessed using BMI in accordance with clinical guidelines [22] and by WC [23]. Abdominal obesity (AO) was diagnosed when WC was > 102 cm in men and > 88 cm in women [23]. The hypertension grade was defined in accordance with the 2023 guidelines of the European Society of Cardiology (ESC)/European Society of Hypertension [24]. Dyslipidemia was defined according to the ESC/European Atherosclerosis Society guidelines for the management of dyslipidemias: lipid modification to reduce cardiovascular risk, 2024 [25]. Diabetes mellitus and impaired fasting glycemia were defined in accordance with the 2022 guidelines of the Russian Association of Endocrinologists for type 2 diabetes mellitus in adults. We assessed impaired fasting glycemia based on questionnaire data and the interpretation of laboratory test results according to the following threshold values: fasting venous blood glucose < 7.0 mmol/L and (if performed) post-oral glucose tolerance test < 7.8 mmol/L [26].

**Table 1:** Clinical Characteristics of the Patients Included in the Study

Parameter	Value (n = 373)
Sex, male/female (%)	38.9/61.1
Age (years)	23 (21; 32)
BMI (kg/m^2^)	23.0 (20.4; 26.3)
Obesity by BMI (%)	10.1
Waist circumference (cm)	
Men	85 (79; 98)
Women	72 (67; 81)
Obesity by waist circumference (%)	14.6
Hip circumference (cm)	
Men	102 (97; 108)
Women	97 (92; 103)
Neck circumference (cm)	
Men	38 (35; 40)
Women	32.5 (31; 35)
Hypertension (%)	15.1
Hypertension duration (years)	1 (0.1; 3.5)
SBP (mm Hg)	126 (119; 135)
DBP (mm Hg)	78 (73; 83.3)
Smoking (%)	30.2
Dyslipidemia (%)	22.8
Total cholesterol (mmol/L)	4.32 (4; 4.9)
LDL-C (mmol/L)	2.3 (2.24; 2.4)
HDL-C (mmol/L)	1.3 (1; 1.5)
TG (mmol/L)	1.0 (0.9; 1.5)
Impaired fasting glycemia (%)	2.4
Glucose (mmol/L)	4.7 (4.3; 5.1)
GFR (mL/min/1.73 m^2^)	98.9 (87.5; 110)
Carotid EMT (mm)	0.43 (0.336; 0.535)
Carotid IMT (mm)	0.7 (0.63; 0.85)

Continuous variables are presented as median (25th; 75th percentile), and categorical variables as percentages. BMI: body mass index; DBP: diastolic blood pressure; EMT: carotid extra-media thickness; GFR: glomerular filtration rate; HDL-C: high-density lipoprotein cholesterol; IMT: carotid intima-media thickness; LDL-C: low-density lipoprotein cholesterol; SBP: systolic blood pressure; TG: triglycerides.

Assessment of the brachiocephalic arteries and measurement of common carotid artery EMT were performed by duplex ultrasound using a Philips Epiq 7DS system (USA, 2018) according to the method described by Skilton et al [27]. Carotid EMT was assessed with a linear transducer at a point 1–1.5 cm proximal to the common carotid artery bifurcation. Carotid EMT was defined as the distance between the inner surface of the posterior wall of the internal jugular vein (relative to the transducer) and the outer surface of the anterior wall of the common carotid artery [27].

IMT was assessed by duplex ultrasound of the brachiocephalic arteries. The results were interpreted as follows: IMT < 0.9 mm was considered normal; 0.9 ≤ IMT < 1.5 mm was considered subclinical atherosclerosis; and IMT ≥ 1.5 mm or focal thickening of 0.5 mm compared with IMT values in adjacent carotid artery segments was considered an atherosclerotic plaque. Patients with atherosclerotic plaques were not included in the study. Measurement of carotid IMT, as well as EMT, was performed bilaterally. Thickness measurements on each side were taken three times, and the mean value was recorded in the study protocol and subsequently used for statistical analysis.

### Statistical analysis

Statistical analysis of the results was performed using Jamovi (version 2.3) software. Variables with a non-normal distribution were presented as the median and the 25th and 75th percentiles. Variables with a normal distribution were presented as the mean with standard deviation. The significance of differences between continuous values was assessed using the Mann–Whitney U test (P (U)). Categorical variables were compared using Pearson’s chi-square test (P (χ^2^)). Spearman’s rank correlation coefficient (ρ) was used to evaluate associations between the studied parameters. Multivariable linear regression analysis was used to assess independent associations between selected predictors and continuous dependent variables, whereas logistic regression analysis was used for binary dependent variables.

For multivariate logistic regression analysis, all continuous independent variables were standardized using the z-transform to obtain an average value of 0 and a standard deviation of 1. Thus, the odds ratios (ORs) represent the change in the probability of developing perivascular obesity (PVO) by the amount of an increase in the corresponding variable by one standard deviation.

To determine the threshold value of EMT, a subgroup of apparently healthy individuals without cardiovascular risk factors, including obesity, hypertension, dyslipidemia, and impaired glucose tolerance (IGT), and impaired fasting glycemia, was selected from the total sample. EMT values exceeding the 90th percentile of the distribution in this subgroup were considered indicative of excessive PVAT deposition, hereafter termed PVO, whereas values below this threshold were regarded as normal EMT.

## Results

The clinical characteristics of the examined sample, including the prevalence of cardiovascular risk factors such as obesity according to BMI, AO, hypertension, smoking, dyslipidemia, and impaired fasting glycemia, as well as the median IMT and EMT of the common carotid arteries, are presented in Table 1.

In the total study sample, the median carotid EMT was 0.43 (0.336; 0.535) mm, whereas the median carotid IMT was 0.70 (0.63; 0.85) mm (Table 1). Both EMT and IMT of the carotid arteries showed an abnormal distribution (Shapiro–Wilk test: P = 0.004 and P < 0.001, respectively) (Fig. 1).

**Figure 1:**
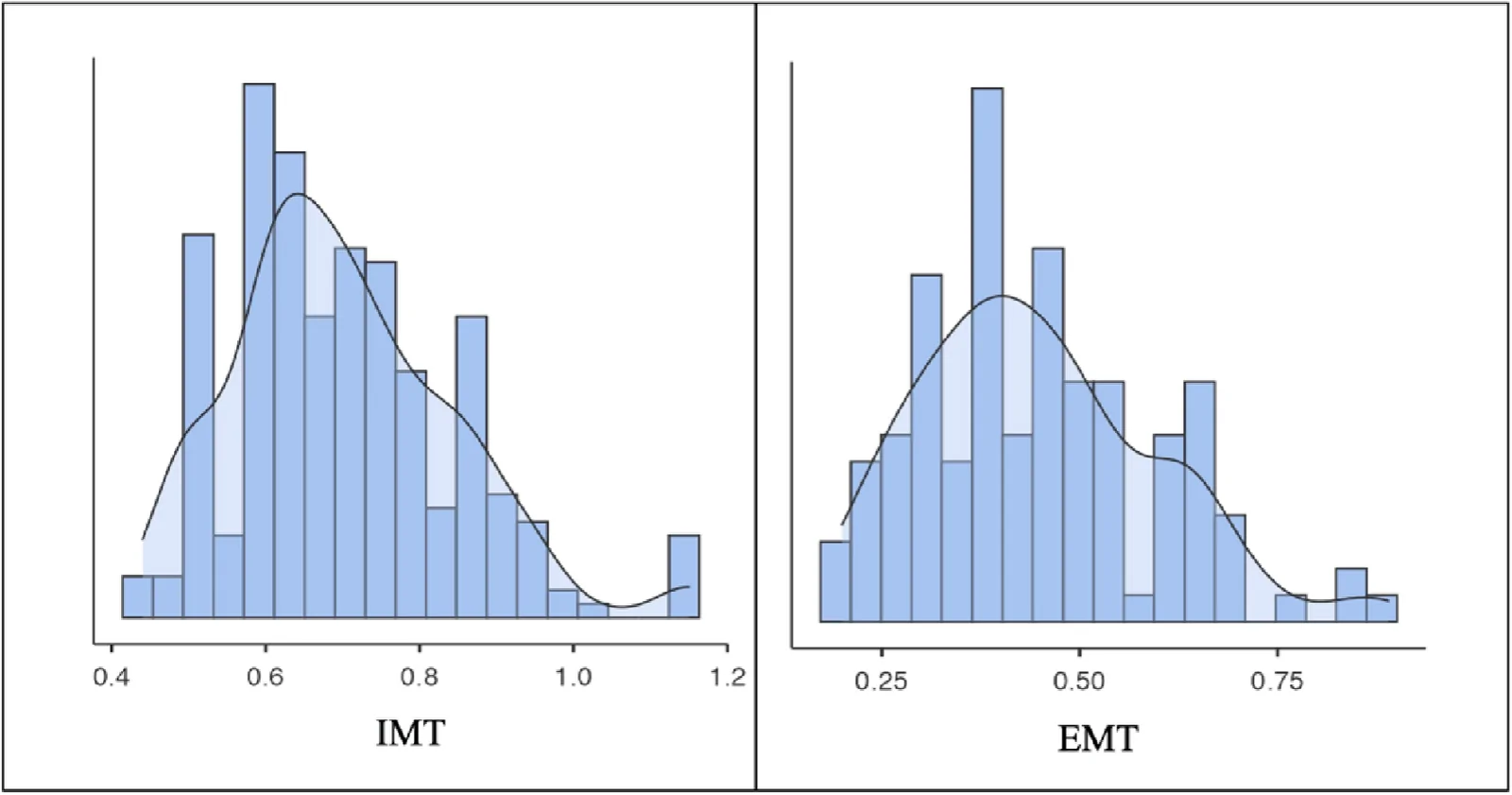
Distribution of the IMT and the EMT of the carotid arteries. EMT: common carotid artery extra-media thickness; IMT: intima-media thickness.

To assess the relationship between carotid EMT and cardiovascular risk factors, univariate correlation analysis was performed. The results are presented in Figure 2.

**Figure 2:**
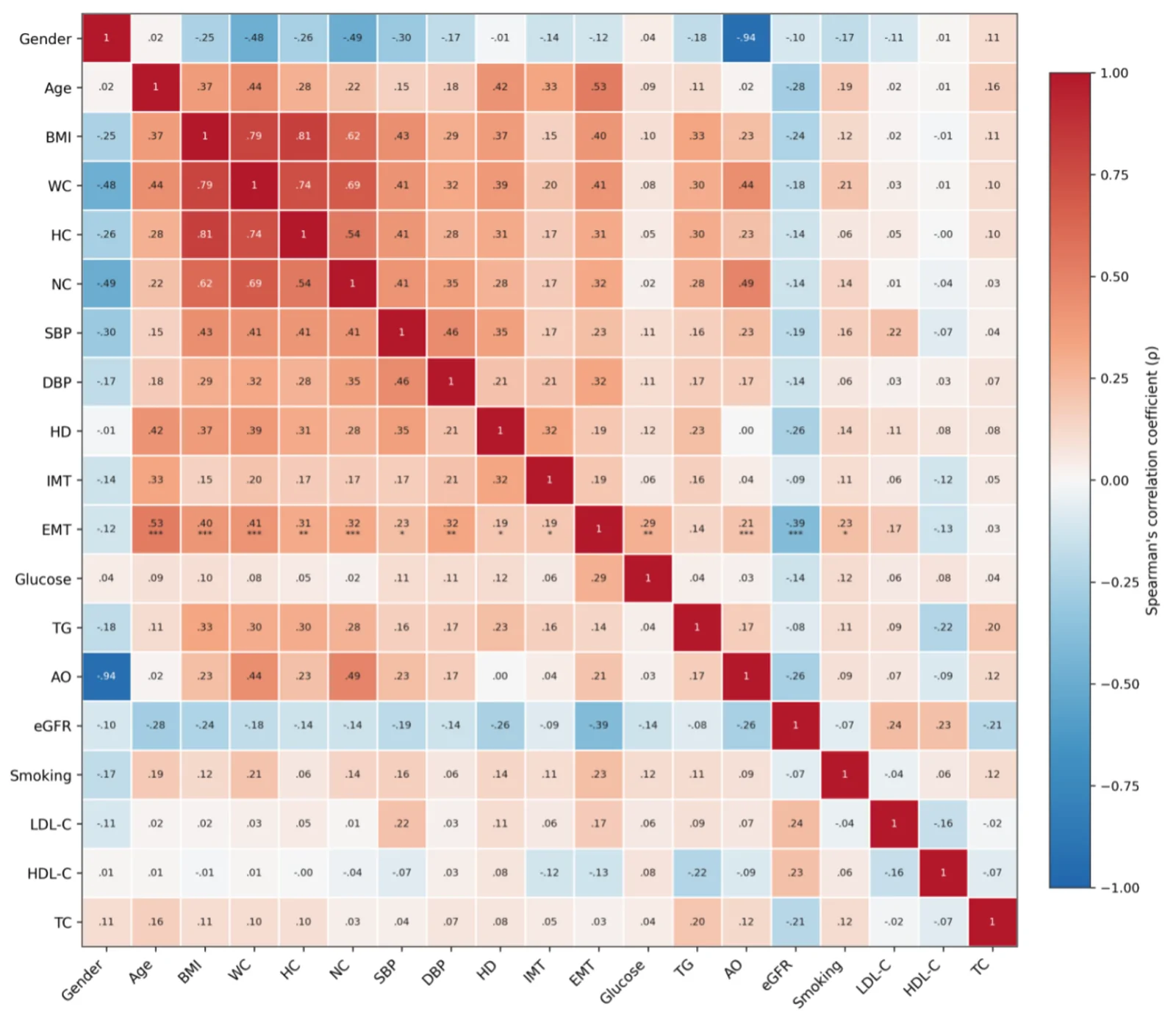
Correlation matrix (heat map) in the examined sample. *P < 0.05; **P < 0.01; ***P < 0.001. AO: abdominal obesity; BMI: body mass index; DBP: diastolic blood pressure; eGFR: estimated glomerular filtration rate; EMT: common carotid artery extra-media thickness; HC: hip circumference; HD: hypertension duration; HDL-C: high-density lipoprotein cholesterol; IMT: intima-media thickness; LDL-C: low-density lipoprotein cholesterol; NC: neck circumference; SBP: systolic blood pressure; TC: total cholesterol; TG: triglycerides; WC: waist circumference.

To assess the independent associations between various factors and EMT, a multivariable linear regression analysis was performed. Carotid EMT was specified as the continuous dependent variable. Models were constructed sequentially. Traditional risk factors (age, sex, obesity, hypertension, smoking, dyslipidemia, impaired fasting glycemia), cardiometabolic risk factors (hypertension duration, smoking duration, blood pressure, BMI, WC, and HC), and biochemical parameters (glycemia, TC, TG, LDL-C, and HDL-C levels) were considered as independent variables. The model with the highest statistical significance is presented in Table 2.

**Table 2:** Multivariable Linear Regression Analysis With Carotid EMT as the Dependent Variable, Adjusted for Age, Obesity, Hypertension, and Smoking

Parameter	Results of regression analysis
Model significance	B = 0.248, SE of B = 0.033, P < 0.001
Age	B = 0.006, SE of B = 0.001, P < 0.001
Obesity	B = 0.219, SE of B = 0.05, P < 0.001
Hypertension	P > 0.05
Smoking	B = 0.053, SE of B = 0.03, P = 0.033

B: regression coefficient; EMT: extra-media thickness; SE: standard error.

To determine a tentative EMT threshold, a subgroup of individuals without cardiovascular risk factors (obesity, hypertension, dyslipidemia, impaired fasting glycemia, and smoking) was selected from the total sample (n = 373). This apparently healthy subgroup comprised 185 individuals, accounting for 49.6% of the total sample. The threshold was defined as the 90th percentile of the EMT distribution in this subgroup. Accordingly, EMT < 0.54 mm was regarded as normal, whereas EMT ≥ 0.54 mm was used as an operational criterion for identifying PVO. Based on this criterion, PVO was identified in 24.2% of patients in the study sample.

PVO was most frequently observed in the group aged 41–55 years and significantly exceeded the prevalence of both BMI-defined obesity and AO (P < 0.001) (Fig. 3).

**Figure 3:**
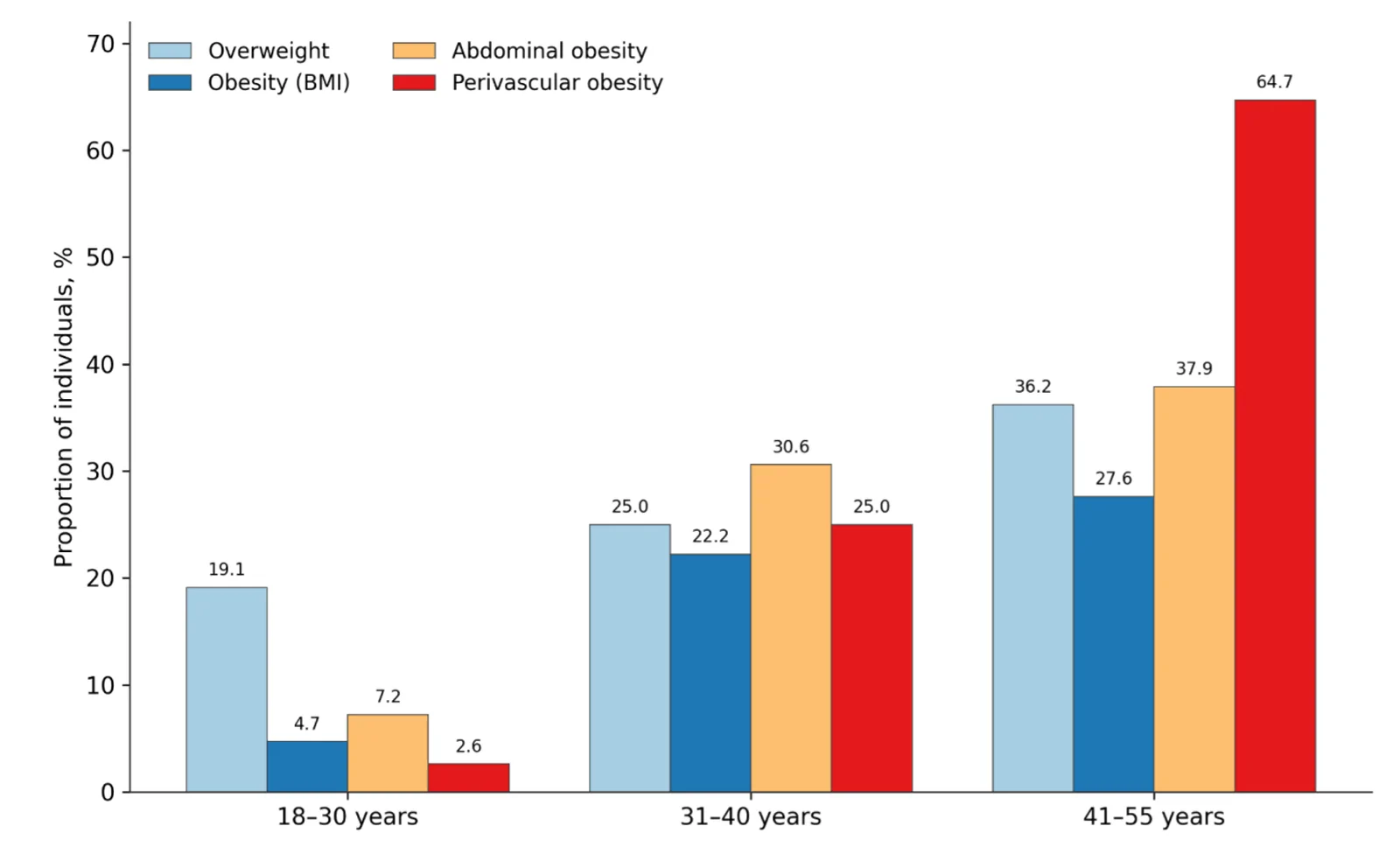
Proportion of individuals with perivascular obesity, overweight, obesity by BMI, and abdominal obesity, by age group. BMI: body mass index.

PVO was detected significantly more often in individuals with obesity defined by BMI and in those with AO than in overweight individuals (P < 0.05) (Fig. 4). Notably, PVO was also identified in individuals with normal body weight (12.9%), suggesting that carotid EMT may identify PVAT accumulation not captured by conventional anthropometric criteria.

**Figure 4:**
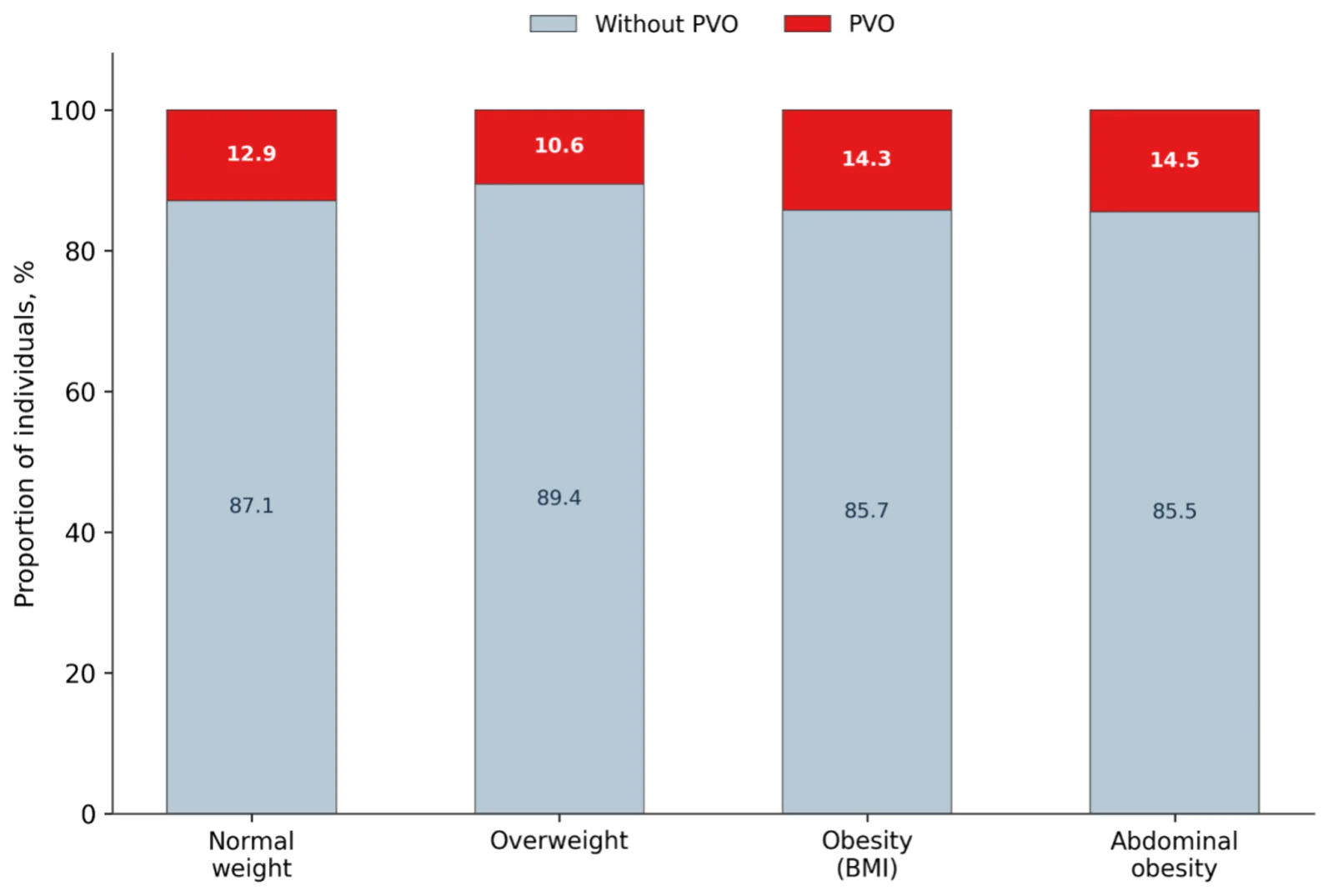
Frequency of individuals with perivascular obesity across groups with normal weight, overweight, obesity by BMI, and abdominal obesity in the examined sample. BMI: body mass index; PVO: perivascular obesity.

To assess independent associations between various CVD risk factors and the presence of PVO, multivariable logistic regression analysis was performed. All continuous independent variables were standardized before multivariable logistic regression analysis. CVD risk factors and their anamnestic characteristics (duration), laboratory characteristics (dyslipidemia and glycemia markers), and instrumental characteristics (systolic blood pressure (SBP), diastolic blood pressure (DBP), and IMT) were sequentially included in the logistic regression models. The final model is presented in Table 3. Age and WC were independently associated with the presence of PVO. A 1-standard-deviation increase in age was associated with higher odds of PVO (OR = 5.22; 95% confidence interval (CI), 2.35–11.55; P < 0.001), as was a 1-standard-deviation increase in WC (OR = 2.82; 95% CI, 1.31–6.09; P = 0.008). Carotid IMT, hypertension duration, TC, and glucose were not independently associated with PVO (Table 3). According to this analysis, age and WC were independently associated with the presence of PVO among individuals without CVD.

**Table 3:** Results of Multivariable Logistic Regression Analysis of the Association of Perivascular Obesity With Risk Factors Using Standardized Continuous Variables

Variable	B	SE of B	P	OR (95% CI)
Age	1.652	0.406	< 0.001	5.22 (2.35–11.55)
Waist circumference	1.036	0.393	0.008	2.82 (1.31–6.09)
IMT	−0.396	0.402	0.324	0.67 (0.31–1.48)
Hypertension duration	−0.744	0.425	0.08	0.48 (0.21–1.09)
TC	−0.231	0.312	0.460	0.79 (0.43–1.46)
Glucose	−0.019	0.375	0.96	0.98 (0.47–2.05)

B: regression coefficient; CI: confidence interval; IMT: intima-media thickness; OR: odds ratio; SE: standard error; TC: total cholesterol.

## Discussion

Carotid EMT is a simple, noninvasive, and clinically accessible ultrasound-based approach for assessing PVAT [28, 29]. Standard examination protocols usually include blood-flow velocity parameters, vessel geometry, IMT, and, when present, the degree of stenosis [30]. In 2009, Skilton et al proposed a method for assessing carotid EMT, which has since been used as an ultrasound marker of PVAT [27].

According to several studies [28, 31, 32], carotid EMT is associated with age-related factors, including the degree of carotid atherosclerotic involvement [29], coronary heart disease [33], and cerebrovascular disease [34].

Our results demonstrated significant associations between carotid EMT and age, blood pressure, anthropometric parameters, laboratory metabolic markers, glycemia, and smoking (Fig. 1). In multivariable analysis, age, obesity, and smoking remained independently associated with EMT (Table 2). No independent association was found between hypertension and EMT, possibly because of the relatively small proportion of participants with hypertension (15%) and the short duration of hypertension in this subgroup.

Lefferts et al demonstrated that carotid EMT was associated not only with markers of visceral adiposity, including WC and sagittal abdominal diameter, but also with vascular stiffness [35]. Unlike their study, which included 135 healthy young men with a mean age of 20 ± 2 years, our study included both men and women across young and middle-aged groups. Similar findings were reported by Carlini et al, who demonstrated significant associations of EMT with age and SBP in a relatively small sample of participants (n = 50; mean age, 42.0 ± 19.0 years) [18].

The adventitial layer is considered a major contributor to increased EMT [36]. The arterial adventitia is a structurally and functionally complex component of the vessel wall that participates in the release and activation of signaling mediators involved in the regulation of vascular tone and homeostasis [37]. Age-related adventitial fibrosis and calcification have been suggested to contribute to the development of arterial stiffness [38]. In addition, dysfunctional PVAT may promote meta-inflammation, impair regional vascular tone and vasa vasorum perfusion, and contribute to increased vascular wall stiffness [37].

According to published data, carotid EMT measured using the method described by Skilton et al [27] was 0.63 mm in individuals with carotid atherosclerosis, 0.38 mm in middle-aged individuals with normal carotid anatomy [31], and 0.80 mm in patients with coronary heart disease [33]. In our study, a tentative EMT threshold of 0.54 mm was identified for increased PVAT in young and middle-aged individuals without CVD. The presence of PVO, defined as EMT ≥ 0.54 mm, was independently associated with age and WC.

Other studies have also demonstrated associations of EMT with AO and its anthropometric marker, WC, as well as with other ectopic fat depots [31]. Druzhilov et al found significant correlations of EMT with both AO and epicardial adipose tissue (r = 0.71, P < 0.001) [31]. In another study involving 100 patients, carotid EMT was associated with epicardial adipose tissue (r = 0.46, P < 0.001) and PAT (r = 0.30, P < 0.001); the mean carotid EMT in that study was 0.66 mm [19].

Our findings suggest that carotid EMT of 0.54 mm or greater may be considered a tentative criterion for identifying PVO. This threshold is within the range of mean EMT values previously reported across different study populations (0.3–0.7 mm) [19, 31, 33]. Further validation of the proposed threshold in various clinical subgroups (including males and females of different age groups, as well as individuals with anthropometric peculiarities, e.g., chest wall deformities [39]) may facilitate the development of novel diagnostic algorithms for stratifying patients according to their risk of CVD and related complications.

Among the limitations of this study, the predominance of young individuals should be noted: 272 participants (73% of the total sample) were younger than 30 years. Owing to the limited sample size, sex- and age-specific differences in tentative EMT reference values as markers of PVO were not assessed. Further studies with larger and more age-balanced samples are required to address these differences.

In conclusion, despite these limitations, carotid EMT was associated with a broad range of cardiometabolic risk factors in individuals without CVD and may reflect PVAT deposition. A tentative threshold of 0.54 mm was identified for detecting possible PVO.

## Data Availability

The data that support the findings of this study are available from the corresponding author upon reasonable request.

## Abbreviations

AO: abdominal obesity
BMI: body mass index
CVD: cardiovascular disease
DBP: diastolic blood pressure
EMT: extra-media thickness
GFR: glomerular filtration rate
HC: hip circumference
HDL-C: high-density lipoprotein cholesterol
IMT: intima-media thickness
LDL-C: low-density lipoprotein cholesterol
MS: metabolic syndrome
MSCT: multislice computed tomography
NC: neck circumference
PAT: pericardial adipose tissue
PRAT: pararenal adipose tissue
PVAT: perivascular adipose tissue
PVO: perivascular obesity
SBP: systolic blood pressure
TC: total cholesterol
TG: triglycerides
WC: waist circumference
